# MALT1 promotes melanoma progression through JNK/c-Jun signaling

**DOI:** 10.1038/oncsis.2017.68

**Published:** 2017-07-31

**Authors:** Y Wang, G Zhang, J Jin, S Degan, Y Tameze, J Y Zhang

**Affiliations:** 1 Department of Dermatology, Duke University Medical Center, Durham, NC, USA; 2 Institute of Dermatology, Chinese Academy of Medical Sciences and Peking Union Medical College, Jiangsu Key Laboratory of Molecular Biology for Skin Diseases and STIs, Nanjing, China; 3 Department of Dermatology, the Fourth Hospital of Hebei Medical University, Shijiazhuang, China; 4 Center for Molecular and Biomolecular Imaging, Duke University, Durham, NC, USA

## Abstract

Mucosa-associated lymphoma antigen 1 (MALT1) is a lymphoma oncogene that regulates signal transduction as a paracaspase and an adaptor protein. Yet, the role of MALT1 in other solid cancers such as melanoma is not well-understood. Here, we demonstrate that MALT1 is overexpressed in malignant melanoma cells, and predicts a poor disease-free survival. MALT1 inhibition via shRNA-mediated gene silencing or pharmacologically with MI-2 compound markedly reduced cell growth and migration of A2058 and A375 melanoma cell lines *in vitro*. Subcutaneous tumor growth analysis revealed that MALT1 gene silencing significantly reduced tumor growth and metastasis to the lung. Consistently, the subcutaneous tumors with MALT1 loss had increased cell apoptosis and decreased proliferation. In addition, these tumors showed signs of mesenchymal–epithelial transition as indicated by the upregulation of E-cadherin and downregulation of N-cadherin and β1-intergrin. Further molecular analysis revealed that MALT1 is required for c-Jun and nuclear factor-κB (NF-κB) activation by tumor necrosis factor-α. Forced expression of the c-Jun upstream activator MKK7 reversed the cell growth and migration defects caused by MALT1 loss. In contrast, NF-κB activation via expression of p65ER, a fusion protein containing NF-κB p65 and the tamoxifen-responsive mutant estrogen receptor, induced minimal effects on cell proliferation, but diminished cell death induced by MALT1 loss and TRAIL treatment. Together, these findings demonstrate that MALT1 promotes melanoma cell proliferation and motility through JNK/c-Jun, and enhances melanoma cell survival through NF-κB, underscoring MALT1 as a potential therapeutic target and biomarker for malignant melanoma.

## Introduction

Melanoma accounts for over 85% of skin cancer deaths. Its incidence has been on the rise at a rate faster than any other cancer in the USA (http://www.cancer.org/cancer/skincancer-melanoma/detailedguide/melanoma-skin-cancer-key-statistics). Once disseminated, metastatic melanoma is resistant to radiotherapy and minimally responsive to chemotherapies comprised DNA alkylating agents such as dacarbazine.^1, 2^ Recently, immunotherapies and oncogene-targeted therapies have expanded melanoma treatment options. Immunotherapies, including IL-2, CTLA-4 inhibitors (e.g. ipilumumab) and PD-1 inhibitors (e.g. nivolumab and pembrolizumab), produce durable remissions in about 5–8% of patients.^2, 3, 4, 5, 6, 7, 8, 9^ The oncokinase inhibitors for BRAF^V600E^ mutant (e.g. vemurafenib and dabrafenib) and MEK (e.g. trametinib and MK164) have increased the response rate to 50% and 20%, respectively.^10, 11, 12, 13^ Nevertheless, the benefit of those therapies is short-lived with many patients succumbing to the disease within 2 years after treatment,^14, 15^ highlighting the need for additional therapies.

Mucosa associated lymphoma antigen 1 (MALT1) regulates nuclear factor-κB (NF-κB) signaling in lymphocytes through two distinct mechanisms.^16, 17, 18^ First, MALT1 is a protein scaffold that recruits TRAF6 to the CARMA–BCl10–MALT1 (CBM) complex.^16^ This recruitment results in the activation of TRAF6 E3 ligase ubiquitin ligase and consequently increased Lys63-linked poly-ubiquitination (K63-Ub) and activation of MALT1, BCL10 and IKKγ.^19, 20, 21^ Second, MALT1 is a paracaspase that cleaves after an arginine residue of a number of NF-κB regulators including BCL10, CYLD, A20, NIK and RelB.^22, 23, 24, 25, 26^ Cleavage of these substrates except BCL10 prevents inactivation of NF-κB signaling.^27, 28, 29, 30^

MALT1 is a driver oncogene in over 50% of mucosa-associated lymphoma and a subset of diffuse large B-cell lymphoma, which is attributed to gene amplification or chromosomal translocation with the apoptosis inhibitor 2 (API2) and immunoglobulin heavy chain loci.^31, 32, 33, 34, 35, 36^ These genetic changes lead to an increased expression of MALT1 or expression of the constitutively active API2-MALT1 fusion protein. Given its high specificity and role in NF-κB signaling, MALT1 has been explored as a favorable therapeutic target for immune defects and cancer.^18, 37, 38, 39, 40, 41, 42^ Of particular interest, recent studies have found that MALT1 is inhibited by several therapeutically active compounds, including MI-2 and the phenothiazine derivatives (mepazine and thioridazine) previously used as dopamine receptor antagonists for psychiatric conditions.^37^ These compounds showed remarkable preclinical efficacies in treating otherwise incurable activated B-cell-like diffuse large B-Cell lymphoma and autoimmune disorders such as multiple sclerosis.^40, 41, 42, 43^

In this study, we sought to examine the role of MALT1 in melanoma. We found that MALT1 is highly expressed in melanoma. Gene silencing of MALT1 markedly slowed melanoma growth *in vitro* and *in vivo*, which was correlated with reduced JNK and NF-κB pathway activation. JNK/c-Jun activation via exogenous expression of a constitutively active mutant of MKK7 reversed cell growth and migration defects. In contrast, NF-κB activation via expression of p65ER reduced cell apoptosis. Our findings demonstrate that MALT1 promotes melanoma progression and survival through JNK/c-Jun and NF-κB signaling pathways, underscoring MALT1 as a potential therapeutic target for melanoma.

## Results

### MALT1 is expressed at an elevated level in malignant melanoma cells

**Figure 1:**
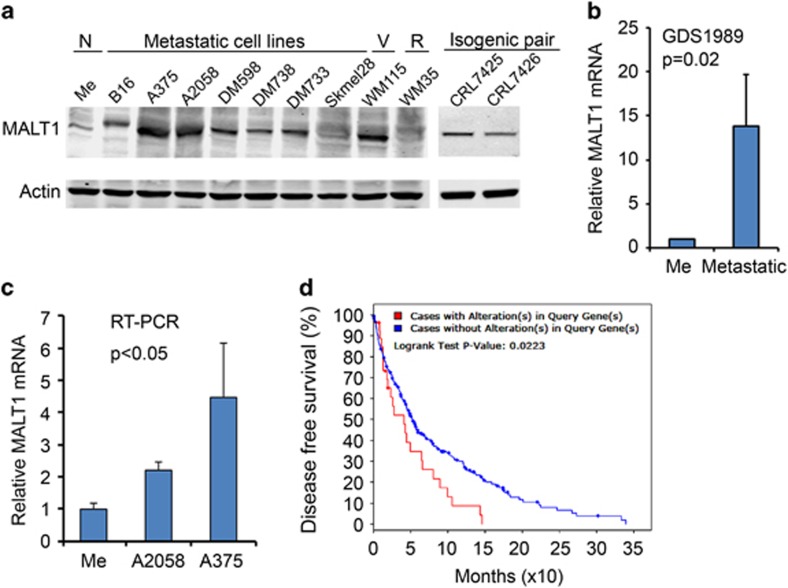
MALT1 is increased in malignant melanoma cells. (**a**) Immunoblotting of protein lysates extracted from normal (N) primary human melanocytes (Me), metastatic, vertical (V) and radial (R) growth melanoma cell lines. (**b**) Relative MALT1 mRNA levels by gene expression profiling (published GEO data sets, GDS1989). (**c**) Relative mRNA levels of MALT1 by real-time reverse transcriptase–PCR with 18 S used as an internal control. Graphs represent averages of relative MALT1 mRNA levels ±s.d. (**d**) Disease-free survival. Data obtained from NCI Cancer Genome Atlas (https://gdc.cancer.gov/)-Skin cutaneous melanoma provisional data. Using (mut and exp>1.1) as search commands in the cBioPortal, 10% of 287 cases showed MALT1 mutation and overexpression.

To establish a clinical relevance of MALT1 to melanoma, we first examined the expression status of MALT1 in melanoma cells. By immunoblotting, we found that MALT1 was expressed at elevated levels in the majority of metastatic melanoma cell lines, including B16, A2058, A375, DM598, DM733, DM788 and CRL7426, as well as CRL7425 (a primary tumor cell line isogenic with CRL7426) and the vertical growth WM115 cells compared with normal melanocytes and the radial growth WM35 cells (Figure 1a). In agreement with protein upregulation, MALT1 mRNA was increased in metastatic melanomas, as shown in two independent gene expression profile data sets (Figure 1b and Supplementary Figures S1a and b). Similarly, quantitative reverse transcriptase–PCR showed that the MALT1 mRNA level was significantly higher in A2058 and A375 cells than in primary melanocytes (Figure 1c). These data indicate that MALT1 is increased at both mRNA and protein levels in malignant melanoma cells. Further analysis of the Cancer Genome Atlas showed that cutaneous melanoma cases with MALT1 gene mutation and mRNA overexpression were associated with a shortened median disease-free survival (Figure 1d).

### MALT1 promotes melanoma cell growth both *in vitro and in vivo*

**Figure 2:**
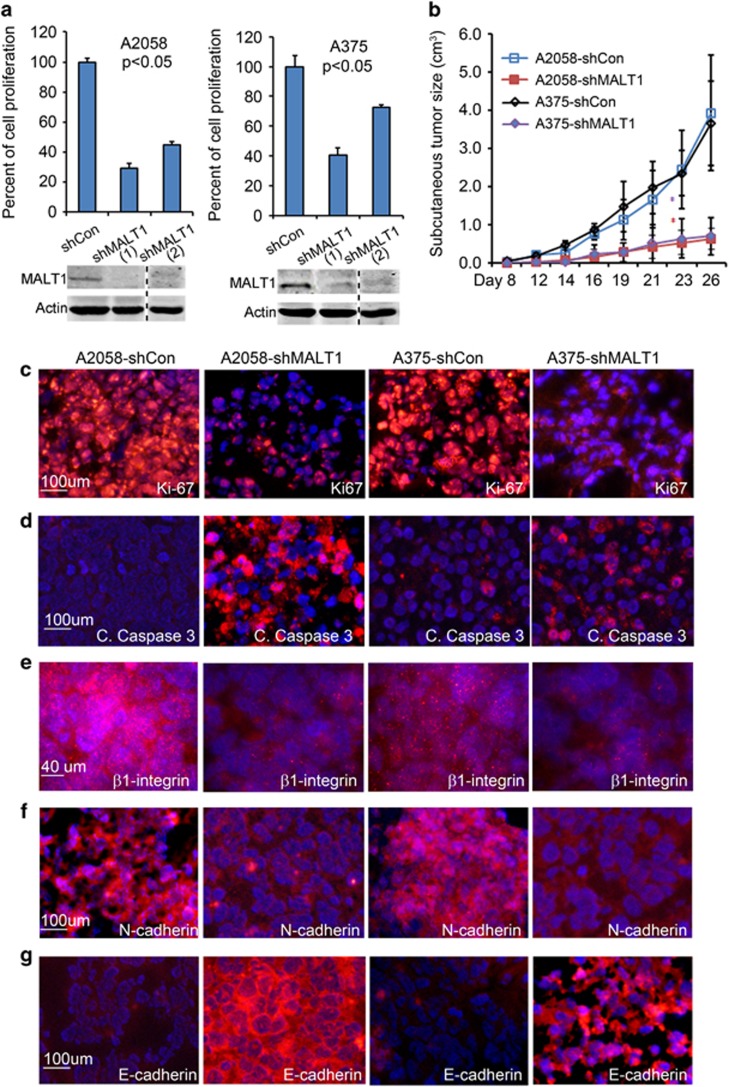
MALT1 gene silencing inhibits melanoma growth and progression. (**a**) Cell growth analysis. A2058 and A375 cells were transduced with lentivirus for expression of non-silencing control (shCon) or two different MALT1-specific shRNA (shMALT1-1 and shMALT1-2), and seeded in triplicates for growth analysis. Graphs represent average percentages of cell growth normalized to control cells ±s.d. *P*-values of less than 0.05 were obtained via two-tiered *T*-test. Efficiency of gene silencing was verified by immunoblotting shown below each graph. (**b**) Subcutaneous tumor growth kinetics. A2058 and A375 cells transduced to expression shCon or shMALT1-1 were injected subcutaneously into immunodeficient NSG mice (*N*=5–10 mice/group with 2 injects/mouse). Tumor sizes were measured biweekly. Graphs represent average tumor size ±s.d. *P-values less than 0.05 were obtained via two-tiered *T*-test. (**c–g**) Immunostaining. Frozen tissue sections were immunostained with primary antibodies against Ki-67, cleaved caspase 3, active β1-integrin, N-cadherin and E-cadherin followed by detection with an Alexa 555-conjugated secondary antibody (orange), nuclei (blue, Hoechst 33528).

To determine whether MALT1 plays an important role in melanoma growth, we first performed shRNA-mediated gene silencing of MALT1 (shMALT1) in metastatic human melanoma A2058 and A375 cell lines through lentiviral gene transduction with two different shRNA constructs. The efficiency of gene silencing was confirmed by immunoblotting (Figure 2a). Cell growth analysis showed that shMALT1 significantly slowed cell proliferation of both cell lines (Figure 2a). Next, we examined melanoma cell growth response to MI-2, a pharmacological agent that inhibits MALT1 function.^40^ By MTT analysis, we found that, while all cell lines responded to MI-2, A2058, A375 and CRL7426 appeared more sensitive than WM35, CRL7425 and SKmel28 (Supplementary Figures S2a and b). These results indicate that MALT1 plays an important role in melanoma cell proliferation and survival, and implicate that metastatic cell lines with high MALT1 expression are especially sensitive to MI-2.

To examine whether MALT1 is important for melanoma growth *in vivo*, we injected A2058 and A375 cells subcutaneously into immunodeficient NSG mice. Cells expressing MALT1 shRNA displayed a significantly reduced tumor growth kinetic as compared with the control cells and consequently produced tumor nodules with significantly lower weight than control tumors (Figure 2b). By immunostaining, we found that the subcutaneous tumors with MALT1 gene silencing were less proliferative and more apoptotic than the control tumors, as indicated by the reduced number of Ki-67-positive cells and the increased number of cleaved caspase 3-stained cells, respectively (Figures 2c and d). To further examine effects on tumor malignancy, we performed immunostaining for β1-integrin, a cell adhesion molecule associated melanoma metastasis.^44, 45^ We found that shMALT1 tumors had markedly reduced β1-integrin expression (Figure 2e). In addition, these tumors exhibited intense immunostaining of E-cadherin (Figure 2f), an epithelial cell marker expressed in normal melanocytes,^46^ and evidently reduced expression of N-cadherin (Figure 2g), a mesenchymal cell marker and promotor of melanoma growth and survival.^47, 48, 49^ Together, these results indicate that MALT1 plays a major role in melanoma growth and malignant progression.

### MALT1 loss impairs melanoma metastasis

**Figure 3:**
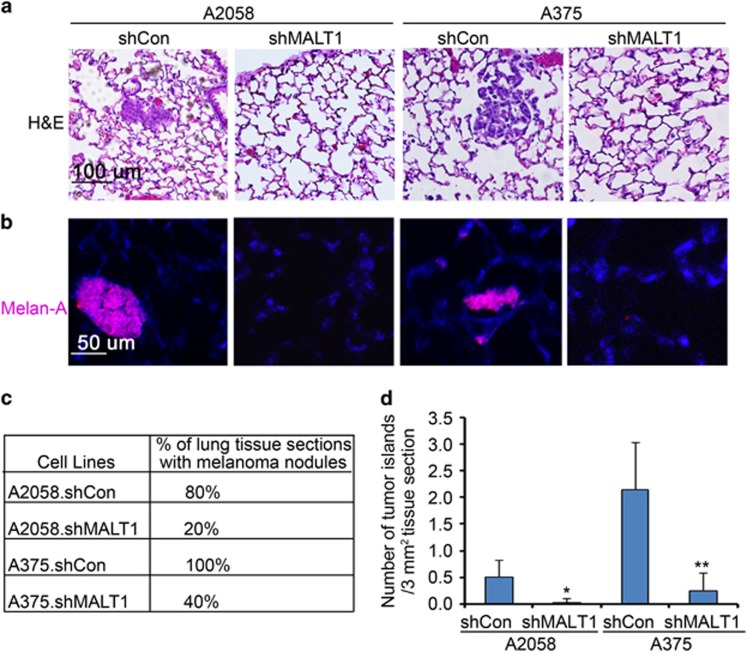
MALT1 loss melanoma metastasis. (**a**) Hematoxylin and eosin staining of paraffin-embedded lung tissues of mice injected with melanoma cells. (**b**) Immunostaining of the lung sections with primary a primary antibody against the melanocyte marker MART-1 (Melan-A) followed by detection with a secondary antibody conjugated with DyLight 549 (orange), Nuclei (blue, Hoechst 33528). (**c**) Percent of pulmonary tissue sections with melanoma cell islands. Ten to 15 tissue sections of each group were examined under a microscope. (**d**) Multiplicity of metastasis. Twelve to 20 images of tissue sections from each animal were examined. Graph represents average number of melanoma cell islands per 3 mm^2^ lung tissue+s.d. Significant *P*-values of (*0.04 and **0.004) were obtained via two-tiered *T*-test between shMALT1 and corresponding shCon groups.

To further assess the effects on metastasis, we collected the pulmonary tissues at the end point. Hematoxylin and eosin staining revealed the compact tumor cell islands in the alveoli tissues derived from the animals injected with control A2058 and A375 cells (Figure 3a). Melanoma origin of these cells was verified by the positive detection of MART-1/Melan-A, a melanocyte-specific marker (Figure 3b). Serial pulmonary tissue section analyses showed that melanoma metastases were readily detected in 80% and 100% of lung tissue sections derived from mice injected with A2058 and A375 control cells, as compared with 20% and 40% of those from mice injected with A2058 and A375 shMALT1 cells, respectively (Figure 3c). In addition, melanoma multiplicity was significantly reduced in shMALT1 groups, as assessed by microscopic quantification of the number of melanoma islands in pulmonary tissue sections (Figure 3d). These results indicate that MALT1 plays a crucial role in melanoma metastasis.

### MALT1 is important for tumor necrosis factor-α induced NF-κB and c-Jun activation

**Figure 4:**
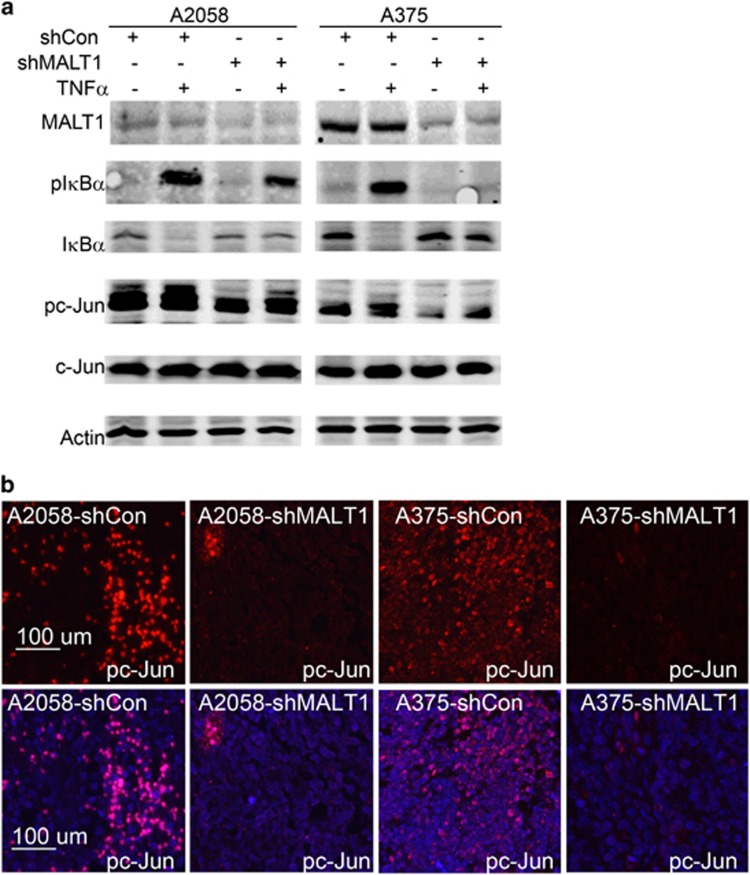
MALT1 is required for NF-κB and c-Jun activation. (**a**) Immunoblotting for pIκBα, IκBα, pc-Jun, c-Jun and Actin. Protein lysates were collected from cells 15 min after treatment with or without TNFα. (**b**) Immunostaining of pc-Jun in subcutaneous tumor samples.

In lymphocytes, MALT1 is a positive regulator of the NF-κB signaling pathway. To test whether MALT1 is required for NF-κB activation in melanoma cells, we treated A2058 and A375 cells with 50 ng/ml tumor necrosis factor-α (TNFα) for 15 min. By immunoblotting, we found that IκBα phosphorylation (pIκBα), a signal for proteosomal degradation of IκBα,^50, 51^ was noticeably induced by TNFα in control cells, but remained low in shMALT1 cells (Figure 4a). Consistently, the total IκBα was reduced by TNFα in control cells but not in shMALT1 cells. A similar pattern of response was observed after treatment with the death ligand TRAIL (Supplementary Figure S3). In parallel to NF-κB, the JNK/AP1 signaling pathway is commonly induced by TNFα, as shown in our previous studies.^44^ Immunoblotting showed that phosphorylation of c-Jun (pc-Jun), a predominant AP1 subunit,^52^ was readily detectable in control melanoma cells, and markedly reduced in shMALT1 cells (Figure 4a). Consistently, immunostaining of the subcutaneous tumor sections demonstrated that nuclear pc-Jun was dramatically decreased in shMALT1 tumors compared with the control (Figure 4b). These findings indicate that MALT1 is required for NF-κB and JNK signaling in melanoma cells.

### Active MKK7 restores melanoma cell growth and migration impaired by MALT1 gene silencing

**Figure 5:**
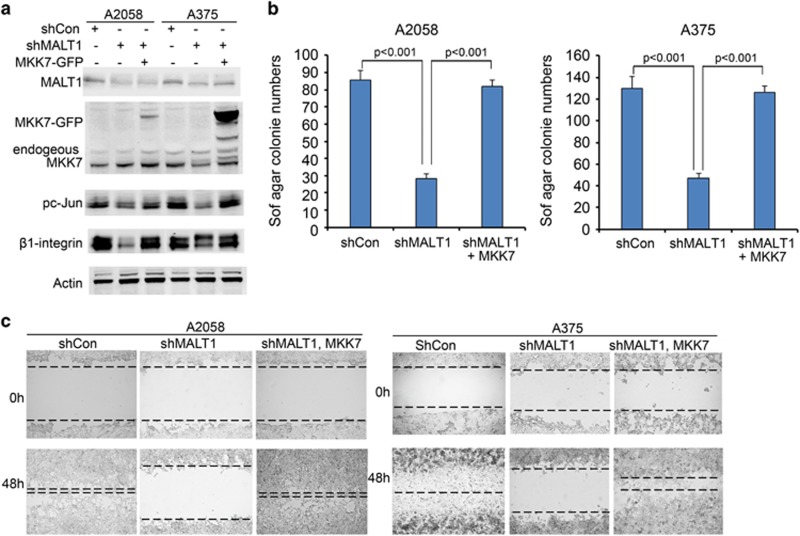
Expression of a constitutively active MKK7 mutant restores melanoma growth and migration. (**a**) Immunoblotting for pc-Jun, c-Jun, β1-integrin and Actin. Protein lysates were collected from A2058 cells transduced for expression of shCon, shMALT1 either with or without the constitutively active MKK7 mutant, and then treated with or without TNFα. (**b**) Soft agar colony assay. Cells transduced as in (**a**) were used for soft agar colony assay, and colony numbers were counted at the 2-week time-point. Graph represents average number of colonies ±s.d. *P*-values of less than 0.05 were obtained via two-tiered Student-T-test. (**c**) Cell migration via scratch-wound assay. Transduced A2058 cells were grown to near confluence, and then incubated with low serum media (0.1% FBS) for overnight and then scratch-wounded. Images were taken at 0  and 24 h time-points.

Next, we asked whether JNK/AP1 signaling is crucial for MALT1-mediated melanoma cell growth. To address this question, we expressed a constitutively active GFP-tagged MKK7 mutant (MKK7-GFP), the upstream activator of JNK, through retroviral-mediated gene transduction as described in our previous studies.^44, 53^ As expected, expression of MKK7-GFP restored c-Jun activation in shMALT1 cells, as verified by immunoblotting for pc-Jun (Figure 5a). Consistently, β1-integrin, a cell adhesion molecule known to be transcriptionally induced by c-Jun in melanoma cells,^44^ was increased by MKK7-GFP (Figure 5a). To determine effects on cell growth, we performed soft agar colony formation, an assay commonly used to assess potential of cancer cell evasion of cell–cell contact induced growth inhibition. Expression of shMALT1 in A2058 and A375 cells resulted in a significantly decreased number of colonies, and expression of MKK7-GFP restored the colony forming ability (Figure 5b). Additionally, shMALT1 markedly slowed cell migration and attachment, and MKK7-GFP fully reversed the migratory defect, as revealed by scratch-wounding and attachment assays (Figure 5c and Supplementary Figure S4). These results indicate that MALT1 acts through the JNK/AP1 signaling pathway to promote melanoma growth and migration.

### NF-κB mediates MALT1-promotion of cell survival

**Figure 6:**
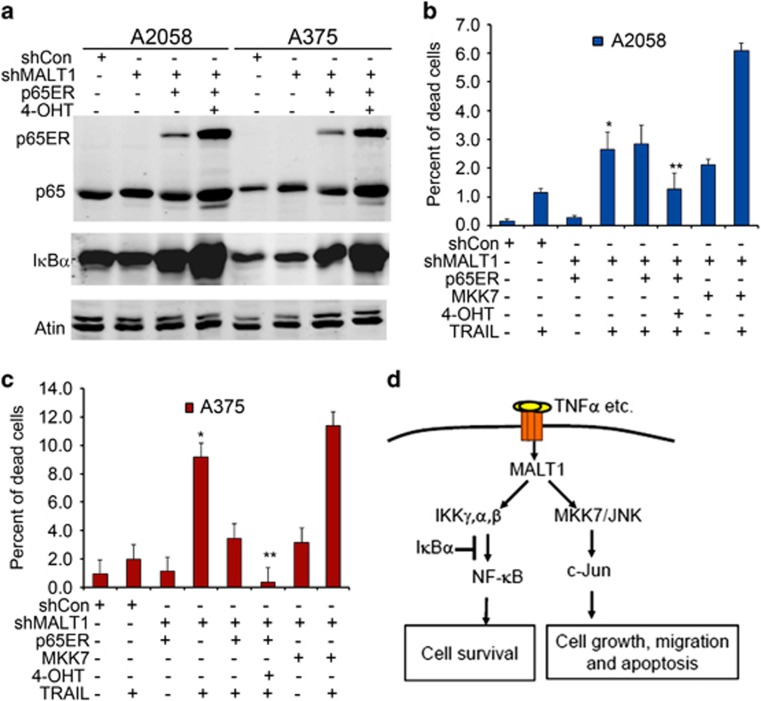
NF-κB activation via 4-OHT inducible expression of p65ER fusion protein prevents cell death induced by MALT1 loss and TRAIL treatment. (**a**) Immunoblotting for p65, IκBα and Actin. Protein lysates were collected from A2058 and A375 cells transduced for expression of shCon, shMALT1 either with or without p65ER, and then treated with 100 nM 4-OHT. (**b**, **c**) Live and dead cell analysis. Cells transduced as in (**a**) were seeded and treated in triplicates with 250 ng/ml TRAIL or 100 nM 4-OHT, and stained with propidium iodide (dead cell) and Hoechst 33285 (live cell) 24 h after treatment with TRAIL. Live and dead cells were counted under a fluorescent microscope as red and blue cells, respectively. Graph represents average percent of dead cells normalized to total cells ±s.d. (**d**) working model.

NF-κB has been characterized as an important regulator of melanoma growth and survival.^54, 55^ We predict that MALT1 loss impairs cell survival due to reduced NF-κB activation. To test this idea, we transduced A375-shMALT1 and A2058-shMALT1 cells the retrovirus encoding p65ER, a fusion protein comprising the predominant NF-κB subunit p65 and the mutant estrogen receptor which is responsive to 4-hydroxytamofen (4-OHT) but not endogenous estrogen.^56^ Inducible expression of p65ER was verified by the increased detection of p65ER and the NF-κB transcriptional target IκBα on immunoblots (Figure 6a). Cell growth analysis showed that p65ER induction via 4-OHT was not effective in rescuing the cell proliferation defects induced by MALT1 loss (Supplementary Figure S5a). Next, we treated cells with the death ligand TRAIL, and then quantified live and dead cells via staining with Hoechst and propidium iodide, respectively. As reported previously,^57^ both A2058 and A375 control cells showed minimal sensitivity to TRAIL-induction of cell death (Figure 6b and Supplementary Figure S5b). MALT1 gene silencing sensitized melanoma cells to death induction by TRAIL, and induction of p65ER with 4-OHT prevented it (Figure 6b and Supplementary Figure S5b). In contrast to p65ER, expression of MKK7-GFP induced an increased rate of cell death in response to TRAIL treatment (Figure 6c). These results indicate that MALT1 acts through NF-κB to promote melanoma cell survival.

## Discussion

Our studies demonstrate that MALT1 is increased in metastatic melanoma at the mRNA and protein levels. Genetic and pharmacological inhibition of MALT1 inhibits melanoma growth and motility. Most importantly, gene silencing of MALT1 markedly reduces subcutaneous melanoma growth and pulmonary metastasis. We further show that MALT1 is required for NF-κB and JNK/AP1 activation. Exogenous expression of the active MKK7 is sufficient to restore melanoma growth and migration in cells with MALT1 loss. Additionally, exogenous induction of NF-κB prevents melanoma cell death sensitized by MALT1 loss. These findings support a working model in which MALT1 promotes melanoma cell proliferation, motility and survival through activation of JNK/AP1 and NF-κB signaling pathways (Figure 6d).

In agreement with our findings, the JNK/AP1 signaling pathway has been implicated in melanoma progression in several other studies. JNK activation is associated with cell proliferation and shorter relapse-free period in superficial spreading malignant melanoma.^58^ Conversely, gene silencing of JNK proteins impairs melanoma cell proliferation, invasion and metastasis, albeit cell line-specific effects were observed.^59^ The JNK/c-Jun signaling cascade also plays a key role in melanoma resistance to therapies, including BRAF/MEK inhibitors, radiation therapy and oncolytic lister strain vaccinia.^60, 61, 62^ In this regard, pc-Jun was detected in the lung metastasis produced by shMATL1 cells (Supplementary Figure S6), which could be a result of regained MALT1 expression or a mechanism contributing to resistance. Moreover, c-Jun stimulates melanoma dedifferentiation and inflammatory cytokine production via counteraction with MITF, resulting in the recruitment of immune suppressive myeloid cells into the tumor microenvironment.^63^ Lastly, JNK/c-Jun inhibition with a peptide inhibitor inhibits melanoma growth in mouse, and attenuates cancer induced pain hypersensitivity.^64^ Thus, JNK/c-Jun promotes tumor cell growth and progression through both cell intrinsic and extrinsic mechanisms. Similar to JNK/AP1, NF-κB has been characterized as potential target for melanoma.^54, 65, 66^ Nonetheless, neither JNK/c-Jun nor NF-κB targeted inhibition has been translated into clinical care for melanoma due to poor pharmacological kinetics or lack of specificity.^67, 68, 69^ Presumably, MALT1 can be explored as an alternative molecular target. In this regard, MALT1 can be inhibited with several medicinally active phenothiazine derivatives including mepazine and thioridazine, the latter of which is previously used as a dopamine antagonist for psychiatric conditions and recently as an antimicrobial agent for tuberculosis.^70, 71^ These agents and the newly characterized MALT1 inhibitor MI-2 have shown efficacy in preventing lymphoma progression in preclinical animal studies.^40, 41, 42^

Together, current findings underscore MALT1 as a promising therapeutic target for melanoma. Future studies may be directed to characterizing the full-spectrum of MALT1 upstream and downstream regulators in melanoma to determine whether MALT1 exhibits any functional dichotomy. It is important to assess the effects of MALT1 inhibition on oncogene-targeted therapies and immunotherapies. The latter is especially important, as MALT1 inactivation in mice results in reduced inflammatory responses and increased autoimmunity due to impaired regulatory T-cell development.^72^

## Materials and methods

### Cell culture and gene transduction

Human melanoma cell lines, including A2058, A375, SKmel28, WM35, CRL7625, CRLD7626 and 293 T cells were obtained from ATCC, and cultured following ATCC recommendations. DM733, DM598, DM738 and DM833 were previously derived from primary biopsies of metastatic melanoma obtained under a Duke University Institutional Review Board (IRB) approved protocol, and kindly provided by Dr Hilliard Seigler (Duke University Medical Center). Cell lines were not authenticated in this study beyond the observation of Melan-A expression. A2058 and A375 were pathogen screened with positive detection of *Myclopasma* sp by PCR and negative for all other pathogens screened (IDEXX BioResearch, Columbia, MO, USA). Other cell lines were not tested. Primary human melanocytes were isolated from surgically discarded neonatal foreskin obtained under a Duke University approved IRB protocol, and cultured in M-254 media (Invitrogen, Carlsbad, CA, USA). The lentiviral MALT1 shRNA plasmid (shMALT1-1) was reported previously,^73^ and the second shMALT1 construct (TRC clone73528) was obtained from Duke RNAi core. The lentiviral plasmid encoding the non-silencing shRNA control was obtained from Invitrogen, and the lentiviral packaging was performed with 293 T cells via co-transfection with psPAX2 and pMD2.G plasmids. LZRS retroviral plasmid encoding constitutively active MKK7 mutant (MKK7(3E)-GFP) were generated and packaged in 293 T phoenix cells as described previously.^53^ Gene transduction of melanoma cells were performed in the presence of 8 μg/ml polybrene, and cells were selected with 1 μg/ml puromycin for about 5 days, as performed in our previous studies.^44^

### Cell growth, adhesion, scratch-wounding and soft agar formation analysis

Cell functional assays were replicated. For cell growth analysis, A2058 and A375 cells (5 × 10^4^) transduced as above were plated in triplicates onto 35-mm dishes and trypsinized for counting under a Zeiss microscope (Jena, Germany) 3 days later. For viability assay, cells were seeded onto 96-well dishes at 10^4^ cells/well, and then treated in hexad with varying doses of the MALT1 inhibitor MI-2 (Selleckchem, Houston, TX, USA) or TRAIL (Genscript, Piscataway, NJ, USA). Three days later, cells were processed for MTT assay with 3-(4,5-dimethylthiazol-2-yl)-2,5 diphenyl tetrazolium bromide (Sigma, St Louis, MO, USA) following the manufacturer’s protocol. For cell attachment analysis, cells were plated onto tissue culture-treated 35-mm dishes, and attached cells were trypsinized for cell counting. Soft agar formation and scratch-wounding analysis were performed as described previously.^44^ Technical variance was similar between each groups in these assay.

### Animal studies

Animal studies were conducted in accordance with protocols approved by the Duke Animal Care and Use Committee. 4 to 6-week-old immunodeficient female NSG SCID mice were purchased from Duke Cancer Center Isolation Facility. For subcutaneous injection, transduced A2058 and A375 cells were selected with 1 μg/ml puromycin for over 5 days, and then trypsinized and suspended at 1 × 10^6^ cells/200 μl injection media containing 150 μl phosphate-buffered saline (PBS) mixed with 50 μl matrigel (BD Biosciences, San Jose, CA, USA). Mice were used in a randomized fashion. Using parameters of *α*=0.05; power⩾80% effect size⩾30% and s.d.⩽20% of the mean, we arrived at a group size of five mice. Tumors were measured biweekly for about 3 weeks with a caliper. The investigators were not blinded during the experiment. At the end point, animals were killed for tissue collection and necropsy.

### Real-time reverse transcriptase–PCR

Total RNA was isolated from melanoma cell lines using Qiagen RNA-isolation kit. Standard real-time reverse transcriptase–PCR for MALT1 was performed with the forward 5′-tgctgttggaagccctattc-3′ and reverse 5′-ccagtaggttccttggtgttc-3′ primers. Reverse transcriptase–PCR for 18 S RNA was performed as an internal control with the forward 5′-tctccggaatcgaaccctgatt-3′ and reverse 5′-cccattcgaacgtctgccctatc-3′ primers.

### Immunoblotting

Whole-cell protein lysates (20 μg each) of primary melanocytes and various melanoma cell lines were separated by electrophoresis on 12% sodium dodecyl sulfate polyacrylamide gel electrophoresis gel, transferred to nitrocellulose membrane and analyzed by immunoblotting with antibodies against MALT1 (#2494), pc-Jun(S73) (#9164), pIκBα(Ser32) (#28590), β1-Integrin (#9699) (Cell signaling Technology, Danvers, MA, USA); c-Jun (Ab-243) (GenScript); RelA/p65 (SC109), IκBα (SC847), MKK7 (SC13071) and Actin (SC16160) (Santa Cruz Biotechnology, Santa CruZ, CA, USA) followed by detection with Alexa IRDye-conjugated secondary antibodies (Invitrogen). Blots were scanned using the Odyssey CLX imaging system (LI-COR, Lincoln, NE, USA).

### Histological analysis

Hematoxylin and eosin staining was performed with paraffin sections by Duke Pathology Laboratory (Durham, NC, USA). Immunostaining of OCT-embedded frozen subcutaneous tumor tissue sections were performed as previously described.^44^ Five-micrometer-thick sections were fixed in 100% methanol for 15 min, blocked with 10% horse serum in PBS (Invitrogen) for 1 h and then incubated with antibodies against Ki-67 (SP6) (Thermo Fisher Scientific, Grand Island, NY, USA); cleaved caspase 3 (#9661), N-cadherin (#13116), E-cadherin (#3195) and β1-Integrin (#9699) (Cell signaling Technology) for 1 h at room temperature or 4 °C overnight. Sections were then washed five times in PBS with 0.05% Tween-20, followed by detection with Alex 555-conjugated secondary antibody (Thermo Fisher Scientific) and counterstaining with 1 μg/ml Hoechst 33258 for 1 min. For immunostaining, paraffin-embedded lung tissue sections were dewaxed in 100% xylene for 5 min followed by sequential 5-min treatments of 100, 90 and 70% ethanol, antigen unmasking by boiling in 10 mM citrate buffer for 10 min and then undergo immunostaining as described above with a primary antibody against Melan-A (HPA048662) (Sigma) and detection with a Dylight 549-conjugated secondary antibody. Fluorescent pictures were taken and processed using the Olympus BX41 microscopic imaging system (Olympus, Center Valley, PA, USA).
