# Supplementary material for: MALT1 promotes melanoma progression through JNK/c-Jun signaling
Source: Oncogenesis. 2017 Jul 31;6(7):e365–. doi: 10.1038/oncsis.2017.68 (PMC5541718; doi:10.1038/oncsis.2017.68)

## Supplementary Figure Legends

**Supplementary Figure S1. MALT1 expression in metastatic melanoma cells.** (**a-b**) MALT1 mRNA is increased in metastatic melanoma. Graphs represent two independent sets of GEO gene expression data (a) GDS1989 [2 samples in each group] and (b) GDS1965, 5 metastatic melanomas and 1 primary tumor].

**Supplementary Figure S2. Pharmacological inhibition of MALT1 reduces melanoma cell proliferation.** (**a**-**b**) Cell growth analysis via MTT assay. Melanoma cell lines, including A2028, A375, WM35, Skmel28, CRL7625 and CRL7626, were seeded in 96-well dishes, and treated in tetrads for 48 hours with varying doses of MI-2. Graph represents average percent of live cells normalized to the untreated group +/- SD.

**Supplementary Figure S3. MALT1 is required for TRAIL-induced NF-κB activation.** Immunoblotting for pIκBα, IκB and Actin. Protein lysates were collected from cells 15 minutes after treatment with or without TNFα.

**Supplementary Figure S4. Active MKK7 rescues melanoma cell attachment.** A2058 and A375 cells transduced to express shCon, shMALT1 either with our without MKK7-GFP were seeded on 35-mm dishes. One hour after seeding, unattached cells were removed together with media, and attached cells were trypisinized for cell counting. Graphs represent average percent of cells attached to the dishes +/- SD.

**Supplementary Figure S5. NF-κB activation via 4-OHT inducible expression of p65ER does not rescue cell proliferation defects, but prevents cell death induction by MALT1 loss and TRAIL treatment.** (**a**) Cell growth analysis. A2058 and A375 cells transduced for expression of shMALT1 and p65ER were treated in triplicates with or without 100 nM 4-OHT for 72 hours. Graphs represent average percentages of cell growth normalized to control cells +/- SD. P-values of less than 0.01 were obtained via two-tiered T-test. (**b**) Representative images of live and dead cells. Cells transduced for expression of shMALT1 and p65ER were seeded and treated in triplicates with 250 ng/ml TRAIL or 100 nM 4-OHT, and stained with propidium iodide (PI) and Hoechst 33285 24h after treatment with TRAIL. Images were taken under florescent microscope. Live and dead cells were identified as red and blue cells, respectively.

**Supplementary Figure S6. pc-Jun expression in lung metastasis.** Immunoflorescent staining of formalin-fixed and paraffin-embedded lung tissue sections with rabbit antibodies against pc-Jun(S73) and mouse antibodies against Melan-A (clone A103, Thermo Fisher Scientific) followed by detection with Dylight-555 [orange] and Dylight-488 [green] conjugated secondary antibodies, respectively. Nuclei, [blue, Hoechst 35228].

Supplementary Figure S1. MALT1 expression is increased in malignant melanoma.

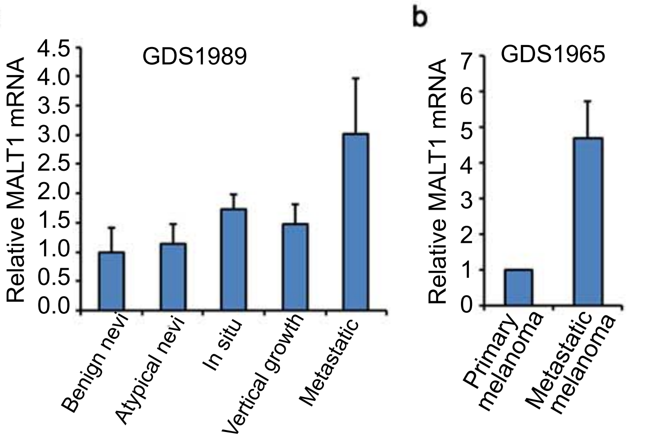


Supplementary Figure S2. MALT1 inhibition reduces melanoma cell proliferation.

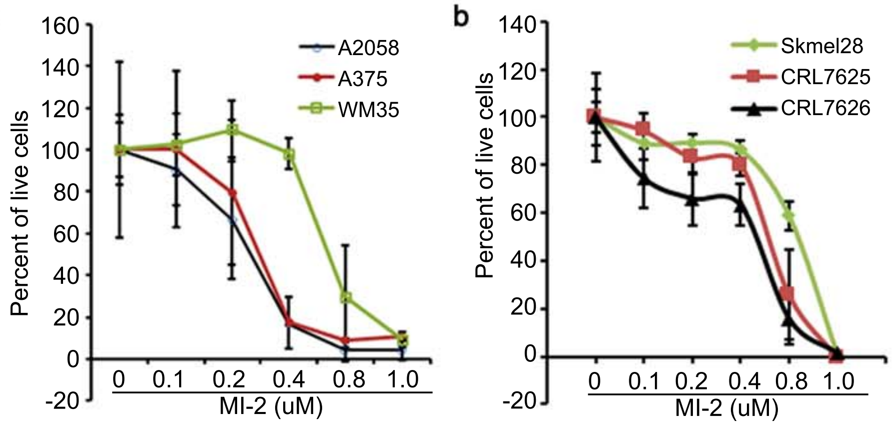


Supplementary Figure S3. MALT1 is required for TRAIL-induced NF-κB activation.

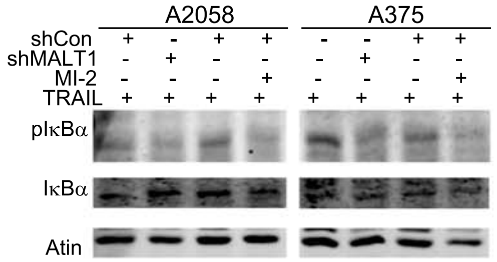

## Supplementary Figure S4. Active MKK7 rescues melanoma cell attachment.

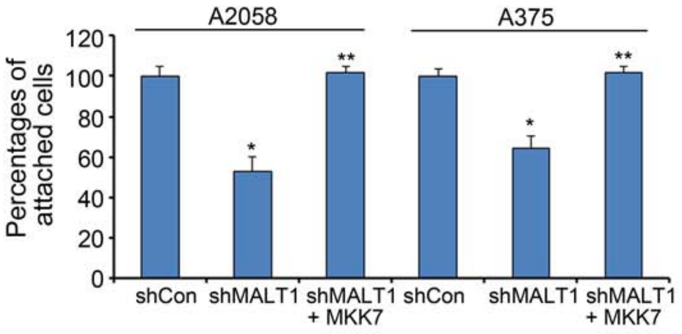


## Supplementary Figure S5. NF-κB activation via 4-OHT-inducible expression of p65ER does not rescue cell growth defects, but prevents cell death induction by MALT1 loss and TRAIL treatment.

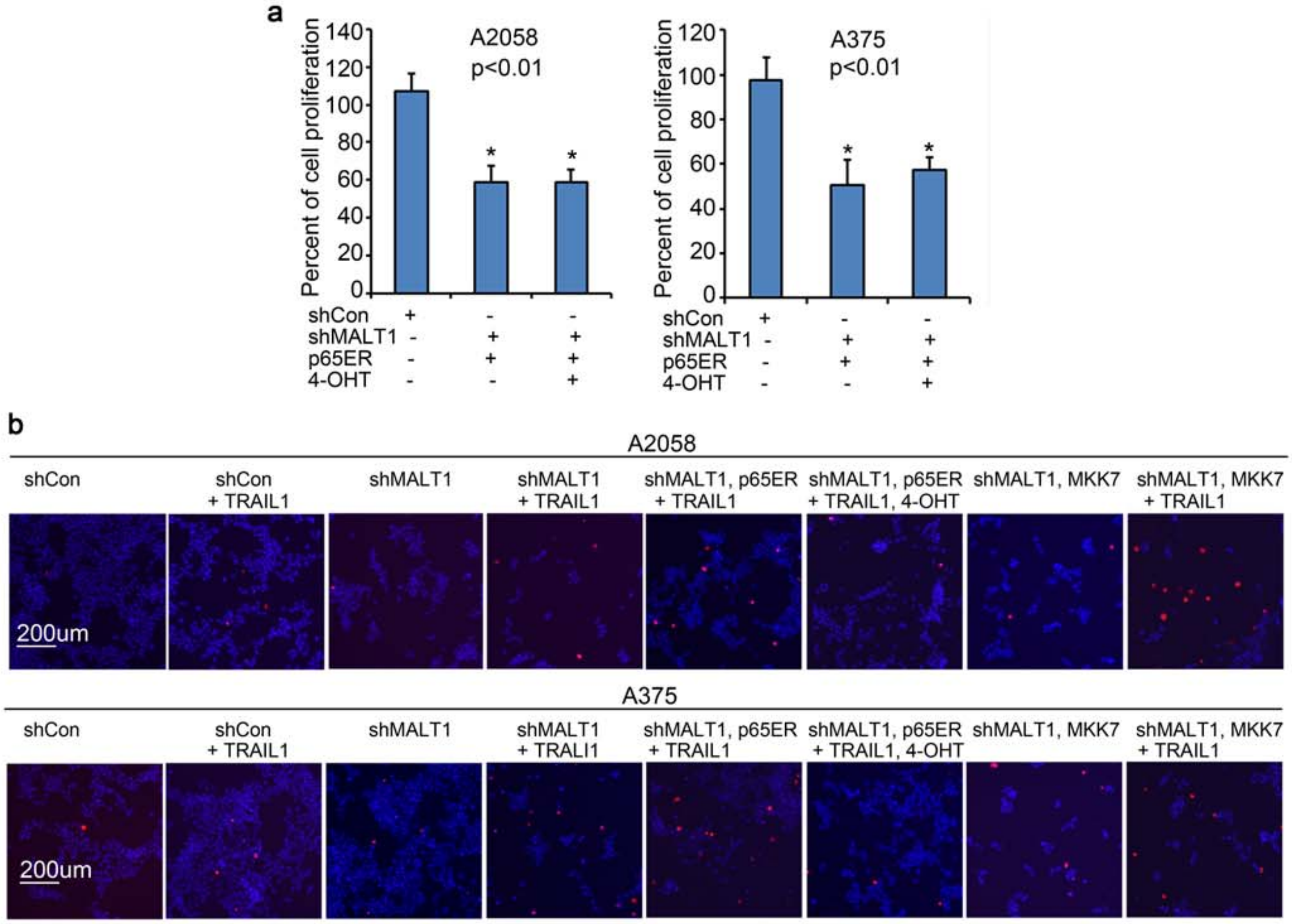

**Supplementary Figure S6. pc-Jun expression in metastatic tumors.**

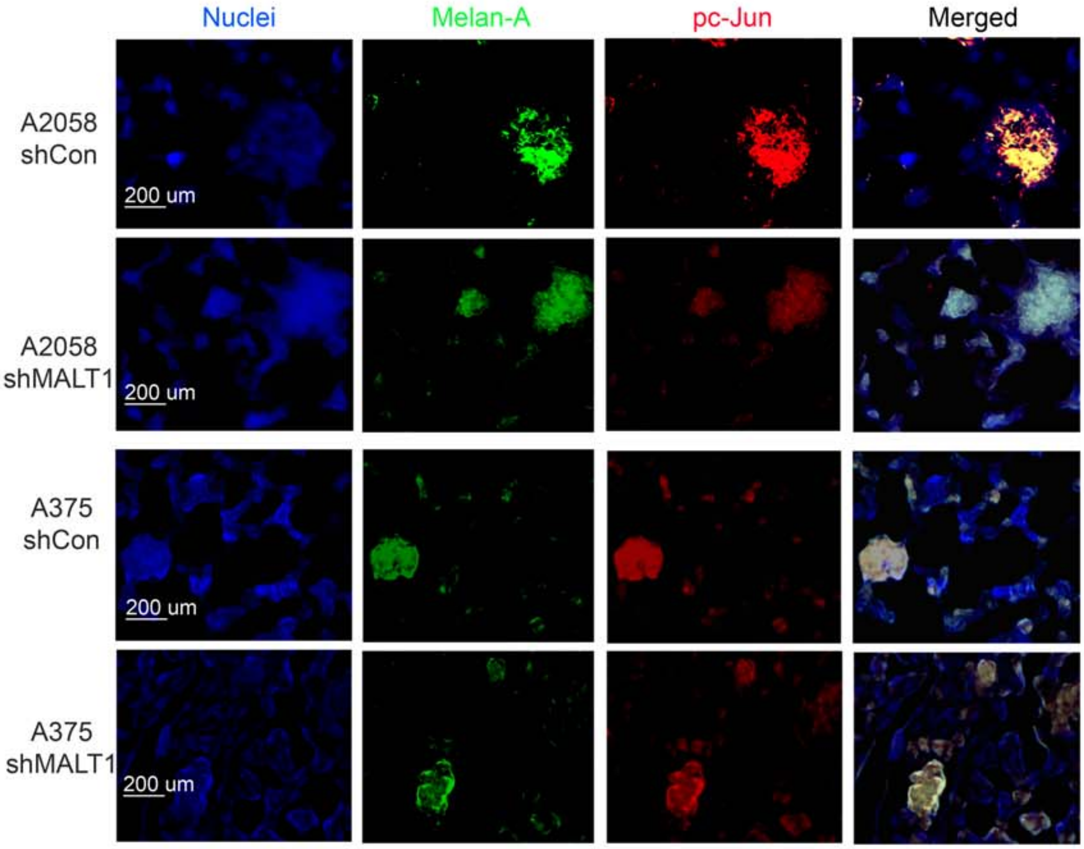

Supplement: Supplementary Figure Legends [file oncsis201768x1.pdf]
